# Supplementary material for: Is Basic Training in Palliative Care Sufficient to Guarantee the Improvement of Knowledge and Skills in This Area?—A Medical Knowledge Assessment Study
Source: Med Sci (Basel). 2025 Sep 2;13(3):167. doi: 10.3390/medsci13030167 (PMC12452678; doi:10.3390/medsci13030167)
Supplement: Supplementary file 1 [file medsci-13-00167-s001.zip › medsci-3796604-supplementary.pdf]

Is basic training in palliative care sufficient to guarantee the improvement of knowledge and skills in this area? – medical knowledge assessment study

Dear colleagues,

At the request of the 6th year student of the Integrated Master's in Medicine at the University of Coimbra, Rita Monteiro, IM3M collaborates in the research project that the student proposed with a view to awarding the Master's degree in Medicine.

The aim of the following questionnaire is to carry out an evaluation and quality improvement study regarding the knowledge of doctors in Palliative Care, with the chosen intervention being the Intensive Course in Palliative Medicine, promoted by the 3M Medical Initiative, between the 20th and 22nd October 2021. The main researcher is student Rita Monteiro, under the guidance of Prof. Dr. Marília Dourado and Dr. Hugo Ribeiro (Faculty of Medicine, University of Coimbra).

The study aims to understand the importance of training in Palliative Care and its application in clinical practice. It also aims to learn about the training offered in the area and its contribution to good medical practice.

To participate, you must be enrolled in the "Intensive Course in Palliative Medicine" organized by the 3M Medical Initiative. The database for researchers to access will be anonymized, with no personal identification appearing in the response to the questionnaire.

This questionnaire will be carried out at 3 different times, before, after and 6 months after the end of the "Intensive Course in Palliative Medicine".

The questionnaire is divided into 3 parts and the average response time is 10 minutes. We request your collaboration, guaranteeing anonymous, confidential and confidential participation. You can stop carrying out the research at any time, without being harmed. The data obtained will be subject to joint statistical analysis, after being placed in an Excel database (for which we request your authorization), without knowing who responded or how they responded. The data will be used exclusively for academic/scientific purposes.

Your participation is extremely important for carrying out this study.

If you have any questions when filling out the questionnaire or require additional clarification, do not hesitate to contact me: monteiro.b.rita@gmail.com

*

☐ I declare that I have read and understood what is contained herein and I freely consent to this study.

Part 1

Sociodemographic Characterization

Gender *

- Feminine
- Masculine

Age *

- 25 to 30 years
- 31 to 40 years
- 41 to 50 years
- 51 to 60 years
- >60 years

Workplace *

- North
- Center
- Lisbon and Tejo's Valley
- Alentejo
- South
- Azores
- Madeira

Years of professional experience: *

- ◯ <5 years
- ◯ 5 to 10 years
- ◯ 11 to 15 years
- ◯ 16 to 20 years
- ◯ 21 to 25 years
- ◯ >25 years

Unit of work *

- ◯ Public hospital
- ◯ Private hospital
- ◯ Primary Care health centers (ACES)
- ◯ Integrated Continuing Care Unit (UCCI)
- ◯ Outra: ______________________

Does your workplace have a Palliative Care Unit or Team? *

- ◯ Yes
- ◯ No
- ◯ I don't know

Do you belong to a Palliative Care Unit or Team? *

- Yes
- No

Are you interested in being part of a Palliative Care Unit or Team? *

- Yes
- No

Do you have prior specific training in Palliative Care (PC)? *

- Yes
- No

If yes, which one?

- Basic (>80 hours)
- Intermediate (between 80 and 180 hours)
- Advanced (>80 hours, masters or doctorate degree)

If not, why?

- ◯ I have no knowledge of training in this area
- ◯ I have no time
- ◯ I'm not interested

Do you have professional experience in Palliative Medicine? *

- ◯ Yes
- ◯ No

If so, what type?

- ◯ Intrahospital Team of Palliative Care Support
- ◯ Community Team of Palliative Care Support
- ◯ Palliative Care Unit/Service in Palliative Care
- ◯ Outra: ______________________

If yes, for how long?

- ◯ < 5 years
- ◯ 5 to 10 years
- ◯ 11 to 15 years
- ◯ >15 years

How would you rate your knowledge in PC? *

- ◯ Insufficient
- ◯ Satisfactory
- ◯ Good
- ◯ Great

Part 2

Agree vs Disagree

Principles of Palliative Care (PC) *

	Agree	Disagree
Even at the end of life, the ethical principle of patient autonomy must be respected.	◯	◯
Early identification and rigorous treatment of problems will not change the patient's prognosis.	◯	◯
PC is offered based on need, not just prognosis or life expectancy.	◯	◯
Patients with pathologies with organ failure do not require PC.	◯	◯
PC should only be considered when no curative therapy is available.	◯	◯
Exclusive concern with symptomatic control is not a good intervention in the patient's suffering.	◯	◯

Referencing Criteria *

	Agree	Disagree
Patients with specific symptoms that are refractory to the best conventional treatment may be referred to PC.	○	○
In terminally ill patients, difficulty in supporting the family member/main caregiver may justify a referral to PC.	○	○
Patients with frequent exacerbations or rapid evolution of the chronic disease should not be referred to PC.	○	○

Gastrointestinal Symptoms *

	Agree	Disagree
The use of a nasogastric tube should be reserved for cases of complete obstructions with abdominal distension.	○	○
It is good PC practice, when prescribing opioids, to also prescribe laxatives systematically.	○	○
Constipation is not a common symptom in PC, therefore, early prevention is unnecessary.	○	○

Respiratory Symptoms *

	Agree	Disagree
Dyspnea not associated with decreased O2 saturation does not need to be addressed.	○	○
Coughing is one of the most unpleasant symptoms for the patient.	○	○
The treatment of cough in patients with CP, whenever possible, should be directed at its etiology.	○	○

Neurological Symptoms *

	Agree	Disagree
Delirium in PC patients is irreversible.	○	○
One of the main etiologies of delirium is the use of morphine.	○	○
As a rule, delirium does not affect memory.	○	○

Communication *

	Agree	Disagree
Communication is one of the best strategies to reduce patient suffering.	○	○
Nonverbal communication encompasses body language and paraverbal language.	○	○
The patient has the right to know about his/her imminent death status if he/she has expressed this wish.	○	○

Suffering *

	Agree	Disagree
When faced with a patient with worsening pain, we must always look for causes of distress that are not physical.	○	○
It is common for a suffering PC patient not to be depressed, as such, the prescription of anti-depressants is not always necessary.	○	○
It is important for a PC patient to maintain a certain level of hope.	○	○

Opioid treatment *

	Agree	Disagree
To reverse delirium in a patient using opioids, it may be effective to rotate opioids.	○	○
The initial dose of morphine in continuous subcutaneous infusion should be equal to the dose of oral morphine.	○	○
After defining the necessary dose of morphine to control symptoms, slow-release formulations can be used.	○	○

Part 3

Practical application

How would you characterize the role of the doctor in a Palliative Care Unit/Team? *

- ◯ Not very relevant
- ◯ Something relevant
- ◯ Very relevant
- ◯ Extremely relevant

In the last 6 months, how many times have you asked a Palliative Care Unit/Team for their opinion? *

- ◯ Not once
- ◯ Few times
- ◯ Sometimes
- ◯ Often

In the last 6 months, approximately, how many patients have you referred to a Palliative Care Unit/Team? *

- ◯ <5
- ◯ 5 to 10
- ◯ 11 to 15
- ◯ 16 to 20
- ◯ >20

What is your level of confidence in referring a patient to a Palliative Care Unit/Team? *

- Very low
- Low
- Average
- High
- Very high

How confident are you in medicating a patient with pain who may be in their last months of life? *

- Very low
- Low
- Average
- High
- Very high

How important is psychological support for caregivers of PC patients? *

- Not important
- Important
- Very important

In the last 6 months, how many times have you referred caregivers of PC patients *
for psychological support?

- ◯ Not once
- ◯ Few times
- ◯ Sometimes
- ◯ Often
